# The Impact of the Alternate Mediterranean Diet (aMED) on the Prevention of Chronic Liver Disease: A Meta-Analysis of Observational Studies

**DOI:** 10.3390/nu17182914

**Published:** 2025-09-09

**Authors:** Linjie Zhang, Jing Sui, Hanlin Yin, Qun Zhao, Yajie Zhou, Hui Xia

**Affiliations:** 1 Research Institute for Environment and Health, Nanjing University of Information Science and Technology, Nanjing 210044, China; zhanglinjieedu@163.com (L.Z.);; 2 Key Laboratory of Environmental Medicine and Engineering of Ministry of Education, Department of Nutrition and Food Hygiene, School of Public Health, Southeast University, Nanjing 210009, China; 3 Jiangsu Provincial Engineering Technology Research Center for Modernization of Traditional Chinese Medicine, Nanjing Zhongke Pharmaceutical Co., Ltd., Zhongke Health Industry Group Co., Ltd., Nanjing 211189, China

**Keywords:** chronic liver disease, alternate Mediterranean diet, dietary patterns, meta-analysis, dietary modifications

## Abstract

Conventional research mainly focuses on individual nutrients or specific foods in relation to chronic liver disease, but the cumulative effects of dietary patterns are underexplored. This study aimed to assess current evidence on the alternate Mediterranean Diet (aMED) and chronic liver disease risk via a meta-analysis of observational studies. A systematic search of PubMed, Web of Science, Cochrane, and Embase (up to February 2025) identified studies on aMED and chronic liver disease, using predefined criteria for screening, quality assessment, and data extraction. A total of 20 articles (3 cross-sectional, 15 cohort, and 2 case–control; 1,286,480 participants) were analyzed using a random-effects model. The analysis showed that aMED was significantly negatively correlated with chronic liver disease (OR = 0.65, 95% CI: 0.56–0.75), indicating that adherence reduces risk. Subgroup analysis showed aMED’s protective effects in North America (OR = 0.67, 95% CI: 0.54–0.83) and Europe (OR = 0.54, 95% CI: 0.44–0.66). The results suggest aMED adherence may lower chronic liver disease risk, emphasizing dietary modifications for prevention.

## 1. Introduction

Chronic liver disease constitutes a significant global health challenge, evident in its extensive patient population, varied clinical presentations, and complex pathological mechanisms [1,2]. Currently, the main pharmacological strategy for chronic liver disease is focused primarily on treating complications and alleviating symptoms, with an emphasis on regulating and improving overall metabolism. In chronic disease management, the principles of “prevention is better than treatment” and “lifestyle modifications complementing pharmacological control” remain crucial [3,4]. It is widely accepted in the academic community that dietary adjustments [5], weight management [6], minimizing sedentary behavior, and regular exercise are effective ways of treating certain chronic liver diseases [4,7].

In recent years, the potential of dietary intervention in the prevention and treatment of liver disease has attracted much attention. Existing studies have revealed various protective factors for chronic liver disease from a nutritional perspective: branched-chain amino acids (BCAAs) can play a liver-protective role through anti-fibrosis and inhibition of hepatocyte apoptosis [8]. Vitamin A analogs slow disease progression through RXR-α-mediated transcriptional regulation, and this conclusion is supported by large cohort studies (*n* = 12,999) [9,10]. The accumulation of lipid droplets (LDs) in hepatocytes exacerbates liver injury through oxidative stress and mitochondrial dysfunction [11]. Studies on food components have also shown that polyphenols exert preventive and therapeutic effects on chronic liver disease. For example, curcumin improves liver fibrosis via the TGF-β pathway, and resveratrol does so via the Hippo pathway [12,13]. Similarly, carotenoids contribute to the prevention and treatment of chronic liver disease; lycopene, for instance, neutralizes reactive oxygen species, while β-cryptoxanthin regulates lipid metabolism [14,15,16]. In terms of individual foods, red meat consumption is positively associated with the risk of nonalcoholic fatty liver disease [17], whereas coffee, blueberries, garlic, and other foods exhibit protective effects [18,19,20,21].

The above studies provide strong evidence of the positive effect of a single nutrient or food on liver disease. However, it is essential to recognize that diet constitutes a complex interplay of various nutrients and other compounds, and individual nutrients or foods exert a synergistic effect within different combinations. This implies the necessity of studying the overall impact of dietary patterns on chronic liver disease.

At present, a substantial body of studies has focused on the role of dietary patterns in the intervention and management of various diseases. Numerous studies have demonstrated that high-quality dietary patterns exert protective effects against a range of conditions, including cardiovascular disease [22], cancer [23,24,25,26], diabetes [27,28], neurological disorders [29,30,31], and immune system disorders [32]. Chronic liver disease typically follows a progressive trajectory characterized by its chronic and insidious nature. This progression creates a critical window for early intervention and control. Dietary interventions possess attributes such as adjustability, multidimensionality, and diverse options. These attributes make them potentially optimal strategies for the prevention and treatment of chronic liver disease.

The Mediterranean dietary pattern, rooted in the traditional dietary patterns of countries bordering the Mediterranean Sea, has gained widespread recognition for its associated health benefits [33]. Evidence from studies indicates that adherence to this dietary pattern is associated with a reduced risk of chronic diseases such as hypertension, type 2 diabetes, and Alzheimer’s disease [34,35]. However, its association with chronic liver disease remains unclear. Accordingly, we searched the relevant literature published prior to February 2025 in the PubMed, Web of Science, Cochrane, and Embase databases, and ultimately selected the alternate Mediterranean diet score (aMED), a high-quality dietary pattern, for analysis. We analyzed its associations with five types of chronic liver disease, specifically, metabolic-associated steatotic liver disease (MASLD, according to the latest disease classification, referred to in this article as the former nonalcoholic fatty liver disease and metabolic-associated fatty liver disease [36]), liver fibrosis, liver cirrhosis, other fatty liver diseases, and liver cancer. Through a comprehensive analysis of the relationship between the aMED dietary pattern and chronic liver disease, we aim to meaningfully supplement and enrich the relevant field.

## 2. Materials and Methods

This study is a meta-analysis of observational studies. The design, implementation, and reporting strictly adhere to the “PRISMA 2020 Statement: Guidelines for Reporting Systematic Reviews and Meta-Analyses” [37]. The study protocol has been registered on the PROSPERO International Prospective Register of Systematic Reviews (Registration number: CRD42025065410).

### 2.1. Data Sources and Searches

To determine the association between aMED and typical chronic liver diseases, we conducted an extensive and systematic search of common terms related to diet, chronic liver diseases, and prevention in four databases: PubMed, Web of Science, Cochrane, and Embase. This study was limited to English-language literature. A total of 12 relevant articles were collected from 2013 to 2025 [38,39,40,41,42,43,44,45,46,47,48,49]

The core search strategy combined “exposure factor (aMED) AND outcome indicator (types of chronic liver diseases)”. Exposure factors included subject terms “Alternative Mediterranean Dietary Score” and “aMED”, connected by “OR”. Outcome indicators included subject terms “Nonalcoholic Fatty Liver Disease”, “NAFLD”, “Metabolic-Associated Fatty Liver Disease”, “MAFLD”, “Metabolic-Associated Steatotic Liver Disease”, “MASLD”, “liver cancer”, “liver fibrosis”, “liver cirrhosis”, and “chronic liver disease”, also connected by “OR”. The exposure and outcome terms were then linked by “AND” to form the basic retrieval framework, thereby ensuring accurate identification of relevant literature on “the association between aMED and chronic liver diseases”. The retrieval formula is as follows.

### 2.2. aMED Dietary Pattern Definition and Validation

aMED (alternate Mediterranean diet score) is an alternative Mediterranean diet scale developed by Fung et al. in 2005, modified from the 2003 traditional Mediterranean diet score (T-MDS), which assesses an individual’s adherence to the Mediterranean dietary pattern [50]. This score includes 9 dietary components: vegetables, fruits, nuts, whole grains, legumes, fish, the ratio of monounsaturated fats to saturated fats (MUFA:SFA), red and processed meat, and alcohol. It ranges from 0 to 9, with higher scores indicating greater adherence to the Mediterranean diet [51].

In a study assessing the reliability and validity of six commonly used dietary quality scores (DQSs), the aMED dietary pattern was validated using a food frequency questionnaire (FFQ). A total of 1394 participants were included, recruited from the Male Lifestyle Validation Study and the Female Lifestyle Validation Study. For reliability assessment, intraclass correlation coefficients (ICCs) of dietary quality scores were calculated from two FFQ administrations (1 year apart) and two 7-day dietary records (7DDRs). For the aMED dietary pattern, the energy-adjusted ICCs were 0.61 in men and 0.62 in women, indicating moderate-to-high test–retest consistency. Validity was verified through comparison with two 7DDRs. Energy-adjusted, attenuation-corrected Spearman correlation coefficients for the aMED dietary pattern were 0.56 in men and 0.62 in women, indicating moderate validity. Furthermore, correlations with plasma biomarkers (e.g., long-chain n-3 fatty acids, carotenoids) aligned with the expected direction, further confirming its biological validity [52].

### 2.3. Study Selection and Eligibility

The selected literature met the following criteria: (1) case–control studies, cohort studies, or cross-sectional studies; (2) studies on the relationship between high-quality dietary patterns and typical chronic liver diseases; (3) reports that provided adjusted hazard ratios (HRs), relative risks (RRs), or odds ratios (ORs) with 95% confidence intervals (CIs); (4) dietary pattern (aMED) as the independent variable, and type of chronic liver disease (NAFLD, MAFLD, MASLD fibrosis, cirrhosis, fatty liver, and liver cancer) as the outcome variable. To avoid terminological confusion, the following clarification is provided: In this study, NAFLD, MAFLD, and MASLD all refer to chronic liver diseases centered on metabolically driven hepatic steatosis. The differences among them are only slight, limited to their naming backgrounds and diagnostic logics. Specifically, NAFLD emphasizes the exclusion of alcohol and other definite liver-damaging factors, with intrahepatic fat deposition as its key feature [53]. MAFLD, defined as metabolic-dysfunction-associated fatty liver disease, requires evidence of hepatic steatosis plus the presence of any one of the following: overweight/obesity, type 2 diabetes, or indicators of metabolic dysregulation (e.g., dyslipidemia, hypertension) [54]. MASLD is an updated term released by international liver disease organizations in 2023; its definition is largely consistent with that of MAFLD. It highlights the central role of metabolic dysfunction in disease pathogenesis and serves as a complement to the NAFLD definition [55].

Exclusion criteria were as follows: (1) animal and cell-related research; (2) letters, interviews, case reports, and comments; (3) less than one year of data collection; (4) studies on acute liver disease; (5) studies without HR, RR, or OR.

### 2.4. Data Extraction

Two researchers (LJ.Z. and J.S.) selected eligible articles based on the title, abstract, and full text of the study. Data on authors, year of study, region, type of study, type of disease, dietary patterns, risk, related indicators, confidence intervals, and confounding variables were extracted. Disagreements in the selection process were discussed with a third researcher (H.X.) to reach a consensus.

### 2.5. Literature Quality Assessment

We assessed the quality of eligible studies using the average Newcastle–Ottawa Scale (NOS) score: above-average studies were defined as high quality, and below-average studies were defined as low quality.

The scoring parameters were as follows: participants could score a maximum of 4 points for the selection criteria, 2 points for comparability, and 3 points for outcome assessment.

### 2.6. Data Analysis

In this study, we extracted data from relevant literature and used OR/RR/HR to indicate effect size. A random-effects model was used to calculate Cochran’s Q and *I*^2^ to assess heterogeneity. If *I*^2^ > 50% and *p* < 0.10, the study showed significant heterogeneity. We conducted meta-regression analyses to check the robustness of the results and further investigate the causes of heterogeneity. A random-effects model was then used, and subgroup analyses were conducted on region, disease type, sample size, and research type to find potential sources. Otherwise, a fixed-effects model was used. Publication bias was assessed using Begg’s funnel plot and Egger’s test. All statistical analyses were performed using Stata 16.0 software.

### 2.7. Pre-Analysis of Heterogeneity Sources

To assess potential sources of heterogeneity, we conducted a pre-analysis to identify factors that might contribute to variability across studies. Heterogeneity was evaluated using Cochran’s Q test and the *I*^2^ statistic, with *I*^2^ > 50% indicating substantial heterogeneity. Potential sources of heterogeneity were hypothesized to include differences in study design (e.g., cross-sectional, case–control, or cohort studies), geographic regions (e.g., Asia, Europe, North America), sample sizes, and disease types (e.g., MASLD, liver fibrosis, liver cirrhosis, liver cancer). Additionally, variations in dietary assessment tools (e.g., food frequency questionnaires, 24 h dietary recalls) and scoring systems for aMED calculation were also considered. To explore these factors, we employed subgroup analyses and meta-regression models, adjusting for covariates (e.g., age, gender, smoking status, physical activity, and medical history). This pre-analysis framework allowed us to systematically investigate and account for heterogeneity, ensuring the robustness and generalizability of our findings.

## 3. Results

### 3.1. Study Screening and Characteristics

**Figure 1:**
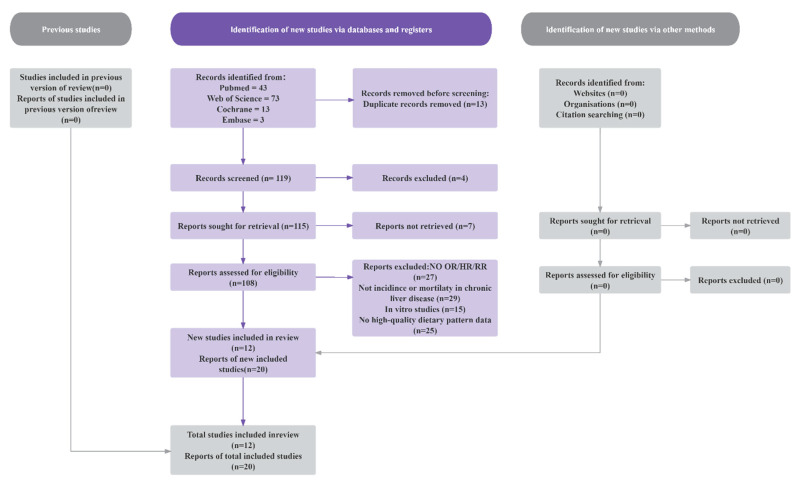
Flow diagram of the study selection.

As shown in Figure 1, we preliminarily selected 12 articles from four databases (PubMed and Web of Science, Cochrane, and Embase), and 20 studies were included as standards in the analysis. As shown in Table 1, these studies were published between 2013 and 2025. The meta-analysis was divided into four different types of chronic liver disease (MASLD, liver fibrosis, liver cirrhosis, liver cancer).

The characteristics of this study are listed in Table 1. Each study included 100–200,000 participants, totaling 1,286,480. The 12 articles included 20 study designs, including 15 prospective cohort studies, 3 cross-sectional studies, and 2 case–control studies. Among them, 5 are from Asia, 5 from Europe, and 10 from North America. After adjusting the data, age, gender, work, smoking status, exercise, and medical history were the main confounding factors.

### 3.2. Quality Assessment

According to the NOS (1 to 9), the scores of the 12 studies ranged from 7 to 9, with an average score of 7.75. Eight studies had higher than average scores and were defined as high-quality studies.

### 3.3. Risk Relationship of aMED for the Prevention of Chronic Liver Disease

**Figure 2:**
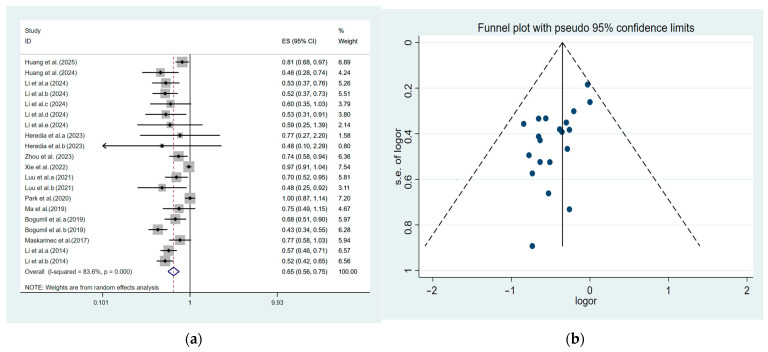
Forest plot and funnel plot for aMED [38,39,40,41,42,43,44,45,46,47,48,49]. (**a**) Forest plot for the association of AMED with chronic liver disease; (**b**) funnel plot for aMED with chronic liver disease. Note: When an article contains multiple research designs, use suffixes such as a, b, c to distinguish these different research designs.

We analyzed the data from the included studies and observed a significant negative association between aMED and chronic liver disease (OR = 0.65, 95% CI: 0.56–0.75) (Figure 2). The result of the random-effects model (*I*^2^ = 0.836, *p* < 0.001) demonstrated significant heterogeneity in this study. We designed four subgroups of disease types, geography, study design, and sample size to explain heterogeneity. Egger’s test (*p* = 0.785) and Begg’s test (*p* = 1.000) indicated that this study had no noticeable publication bias.

### 3.4. Sensitivity Analysis

To comprehensively explore the potential association between aMED and chronic liver diseases, and to assess the stability and reliability of the findings, we conducted sensitivity analyses of the relevant literature.

In the sensitivity analysis of 20 studies exploring the relationship between aMED and chronic liver disease, the overall result was OR = 0.65, 95% CI: 0.56–0.75. Among these studies, the study by Park et al. likely had the greatest impact on the overall result [45]. In addition, two other studies may also have prominently influenced the overall results [43,47]. The results after exclusion of each of them were consistent with the overall result (OR = 0.63, 95% CI: 0.53–0.74; OR = 0.67, 95% CI: 0.58–0.77; OR = 0.63, 95% CI: 0.55–0.73).

The results of all the above analyses showed that all the included studies had a high degree of consistency and reliability, and the corresponding conclusions were persuasive and generalizable.

### 3.5. Meta-Regression

Random-effects model data indicated significant heterogeneity in studies investigating the relationship between aMED and chronic liver disease (*I*^2^ = 0.836, *p* < 0.001). We assessed the impact of covariates such as disease type, geographical location, study design, and sample size on effect sizes through meta-regression analyses. The results indicated that in aMED studies, disease type (e.g., MASLD, liver fibrosis, liver cirrhosis), geographical location (e.g., Asia, Europe, North America), and study design (e.g., cross-sectional studies, cohort studies) were significant sources of heterogeneity (*p* < 0.05).

### 3.6. Subgroup Analysis

The model selection for subgroup analysis in this study was based on the following principles: in addition to referring to the results of the *I*^2^ statistic and Q-test, the random-effects model was consistently employed for two types of subgroups: low-heterogeneity subgroups with differences in population baselines or study designs, and single-study subgroups where heterogeneity could not be tested. This approach aimed to reduce bias caused by clinical or methodological variations.

In the results of the meta-regression analyses, some of the covariates were not significant for effect sizes but may still influence the relationship between dietary patterns and chronic liver disease in different combinations or in specific contexts. To explore this potential association and sources of heterogeneity in more depth, we built subgroups for analysis around disease type, geography, study design, and sample size. This approach provides a richer perspective for a comprehensive understanding of the relationship. 

Subgroup analysis of the disease types (Table 2) showed that aMED was significantly and negatively associated with MASLD (OR = 0.86, 95% CI: 0.75–0.99), chronic liver disease (OR = 0.52, 95% CI: 0.46–0.58), and liver cancer (OR = 0.69, 95% CI: 0.60–0.79), while not significant for liver fibrosis (OR = 0.48, 95% CI: 0.10–2.29). The regional subgroup analysis revealed a significant inverse correlation between aMED and chronic liver disease in Asia (OR = 0.70, 95% CI: 0.53–0.92), North America (OR = 0.67, 95% CI: 0.54–0.83), and Europe (OR = 0.54, 95% CI: 0.44–0.66). In study design subgroups, cohort (OR = 0.66, 95% CI: 0.55–0.78), case–control (OR = 0.47, 95% CI: 0.32–0.69), and cross-sectional (OR = 0.81, 95% CI: 0.68–0.96) showed that aMED was negatively associated with the risk of chronic liver disease. In subgroups by sample size, we observed a significant negative association between aMED and chronic liver disease in groups with >1000 participants (OR = 0.50, 95% CI: 0.33–0.76) and in groups with <1000 participants (OR = 0.66, 95% CI: 0.57–0.77).

## 4. Discussion

At present, there are numerous studies in relevant fields exploring the relationship between dietary patterns and chronic liver diseases. However, most of these studies only focus on the impact on a specific type of chronic liver disease and lack a systematic analysis of the alternate Mediterranean diet score (aMED) across various chronic liver diseases. After extensive data collection, we focused our research on aMED, a widely recognized high-quality dietary pattern, to systematically investigate its association with multiple chronic liver diseases.

In this study, aMED showed a significant preventive effect on chronic liver diseases. To our knowledge, this is the first comprehensive meta-analysis specifically exploring the relationship between aMED and the risk of multiple chronic liver diseases. The results indicated that adherence to the aMED pattern can significantly reduce the risk of developing chronic liver disease (OR = 0.65, 95% CI: 0.56–0.75), which is consistent with the conclusions of previous relevant reviews.

Compared with previous studies focusing on aMED, this study included 12 articles published from 2013 to 2025, covering 20 eligible study items and involving 1,286,480 participants from multiple regions. This larger sample makes our study more statistically valid and the results more convincing.

Studies have already found that a poor diet high in saturated fats, sugar, and calories can lead to a variety of negative consequences in the body, such as fatty acid accumulation, oxidative stress, and the triggering of inflammation [56,57,58,59,60]. These consequences promote the development and progression of chronic liver diseases. A healthy diet, on the other hand, has a positive effect on chronic liver diseases. Combining the characteristics of the aMED dietary pattern and the main causes of the emergence and development of chronic liver diseases, we propose several mechanisms to explain the relationship between the aMED dietary pattern and the prevention of chronic liver diseases.

Fat accumulation is an important factor in the development of chronic liver diseases [61]. The aMED dietary pattern emphasizes the intake of vegetables and whole grains. Within this dietary framework, dietary fiber contributes to enhanced satiety, which in turn helps regulate total calorie consumption [62]. Concurrently, dietary fiber facilitates the production of short-chain fatty acids (SCFAs) by gut microbiota, directly inhibiting lipid synthesis in the liver [63]. High-quality proteins derived from fish and legumes can downregulate the expression of key enzymes involved in lipid synthesis, specifically fatty acid synthase (FASN) and stearoyl-CoA desaturase 1 (SCD1), and also affect (DIT) [64,65,66]. Inflammation is a key driver of chronic liver disease progression. A diet rich in saturated fats and refined sugars readily activates the liver’s innate immune system [67]. Notably, multiple components of the aMED dietary pattern exert anti-inflammatory effects. Vitamin C from oranges and tomatoes, along with flavonoids from blueberries, helps reduce inflammatory factors [68,69,70], and omega-3 fatty acids from fish inhibit the production of pro-inflammatory mediators (e.g., prostaglandin E2, leukotriene B4) [71]. Oxidative stress is the main cause of chronic liver diseases. Carotenoids from tomatoes can activate antioxidant enzymes, thereby mitigating redox imbalance [72]. Vitamin E in nuts helps alleviate free radical-induced cellular damage [73]. Additionally, docosahexaenoic acid (DHA) from fish enhances the activity of glutathione peroxidase and suppresses the excessive production of reactive oxygen species (ROS) [74,75]. Dysregulated food intake is one of the main causes of insulin resistance [76]. A high-quality dietary pattern may improve insulin resistance. Dietary fiber from whole grains contributes to improved insulin secretion [77,78]. Unsaturated fats in vegetable oils help enhance insulin sensitivity [79]. Resveratrol in red wine exerts a protective effect on pancreatic islet β-cells [80,81]. Dietary diversity is a core characteristic of high-quality dietary patterns [82], and its protective effect against chronic liver disease primarily manifests in two aspects. First, the intake of a wide range of nutrients supports the proliferation of beneficial gut bacteria and helps maintain gut microbiota balance. For instance, polyphenols can induce beneficial bacteria to produce metabolites like SCFAs and promote the secretion of signaling molecules such as serotonin and γ-aminobutyric acid to regulate the microbiota [83]. Second, diverse diets exhibit synergistic effects among food components: for example, the combination of polyphenols and vitamin C reduces inflammatory responses [84]. The combination of fish oil and vitamin D lowers insulin levels in patients with NAFLD [85]. Vitamin C enhances the protective effect of potassium against NAFLD [86]. This multi-component synergy provides important support for the aMED dietary pattern to exert its hepatoprotective effects.

This study includes 12 articles with diverse research designs, namely cross-sectional, case–control, cohort, and prospective studies. Results from different designs corroborate and complement each other, enhancing the comprehensiveness and reliability of the overall findings. The included studies involve a cumulative 1,286,480 general population sample, supporting the generalizability and replicability of the results.

To our knowledge, this is the first article to systematically assess the relationship between aMED and the risk of chronic liver disease, diverging from past research that focused on the impact of individual nutrients, foods, and dietary patterns on the disease, and providing a more comprehensive and realistic guide. We performed multiple subgroup analyses for heterogeneity to increase the robustness of the findings. In this study, sensitivity analysis was employed to confirm consistency among the included studies. The meta-regression model was used to account for potential heterogeneity in multiple subgroup analyses to enhance generalizability.

In the subgroup analyses, certain associations between dietary patterns and chronic liver diseases did not reach statistical significance. The aMED diet did not demonstrate a significant association with liver fibrosis (OR = 0.48, 95% CI: 0.10–2.29). These non-significant findings may be attributed to several factors. First, the limited sample size in certain subgroups (e.g., studies focusing on liver fibrosis or cirrhosis) may have reduced statistical power to detect significant associations. Second, regional variations in dietary habits and disease prevalence could influence the observed effects. For example, the aMED diet, which is rooted in Mediterranean eating habits, may exhibit stronger protective effects in Mediterranean populations compared to other regions. Third, differences in study designs (e.g., cross-sectional vs. cohort studies) and dietary assessment tools may introduce heterogeneity, potentially diluting the observed associations.

There are some limitations to this study. Firstly, in terms of data, this study covered a variety of research designs. Due to the different measurement tools and evaluation methods used in the included studies, the data may be heterogeneous. Specifically, different dietary assessment tools differ in accuracy, coverage, and emphasis on the assessment of eating habits. Although some confounding factors were controlled through covariate adjustment and subgroup analysis, residual uncontrolled factors (e.g., unmeasured lifestyle, genetic susceptibility, socioeconomic status) cannot be excluded and may overestimate the Mediterranean diet’s hepatoprotective effect. Additionally, variations in dietary pattern definitions and scoring criteria across studies affect data comparison and integration accuracy. This study did not directly pool effect sizes, so potential bias from variations in effect size types cannot be fully ruled out. Secondly, in terms of research design, each included design has inherent drawbacks. Case–control studies may be subject to recall bias. Cross-sectional studies struggle to infer causality, fail to reflect dynamic changes, and introduce recall bias via participant-dependent recollection. Cohort studies observe temporal exposure–outcome relationships but may incur bias from loss to long-term follow-up, diminishing the reliability of results. These design limitations introduce heterogeneity to the study. Furthermore, differing external validity across designs makes it hard to determine the applicable population and scenarios for combined results. Thirdly, in terms of methodology, this study used meta-analysis to integrate studies and draw general conclusions, but these conclusions risk overgeneralization due to overlooked individual differences and special cases. Additionally, observational studies have inherent limitations. Reverse causality may exist (e.g., early asymptomatic chronic liver disease could prompt dietary changes, leading to the observed association between “high Mediterranean diet adherence” and “lower liver disease risk”), and no mediation effect analysis was performed, precluding confirmation of a direct causal link between the Mediterranean diet and reduced liver disease risk. Finally, this study included multi-regional research designs. However, regional dietary cultural differences may limit the conclusions’ generalizability, specifically in terms of variations in compatibility between local dietary traditions and the aMED, food ingredient substitutability, and cooking methods.

In view of the limitations of this study, we will address the following aspects in future research. We will construct a multivariate model to refine and analyze more variables, thereby exploring the association between multiple factors and chronic liver diseases in greater depth. In the future, it is essential to conduct studies with larger sample sizes, adopt standardized dietary assessment methods, and perform region-specific analyses. This will clarify the impact of potential heterogeneity introduced by differences in study designs and dietary assessment tools on research results and further explore potential influencing factors. Considering the sample size, external validity, and other aspects of the study design, future studies should assign appropriate weights to the included studies. This would ensure that the contribution of each study is accurately reflected when aggregating results, thereby enhancing the applicability and robustness of the conclusions. Additionally, we will verify the findings with actual cases to enhance the precision of the conclusions and promote progress in this field.

## 5. Conclusions

In summary, significant associations have been identified between aMED and various chronic liver diseases. The findings suggest that adherence to healthy dietary patterns may lower the risk of developing such diseases. These results underscore the potential of dietary modifications in the prevention and management of chronic liver diseases.

This study overcomes the limitations of single-factor research and provides a new perspective on chronic liver disease intervention, potentially facilitating evidence-based strategies in disease prevention. Nevertheless, the efficacy of dietary patterns in the prevention of chronic liver diseases is affected by a variety of factors, including geography, degree of illness, and physical activity. We will further explore the preventive mechanisms of dietary patterns for chronic liver diseases to provide more detailed guidance for their management.

## Figures and Tables

**Table 1:** General characteristics of the included studies.

Study	Disease	Location	Specific Countries	Study Design	Sample Size	Adjustment Variables	NOS Score
Huang et al. (2025) [38]	NAFLD, MASLD	North America	USA	cross-sectional	70,190	age, sex, race, BMI, hypertension, and diabetes	7
Huang et al. (2024) [39]	MAFLD, MASLD	Asia	China	case–control	456	age, education level, monthly household income, marriage, physical activity, smoking, and total energy intake	8
Li et al. (2024) [40]	chronic liver disease, liver-related death, liver cancer, hepatocellular carcinoma	Europe	UK	cohort	459,502	age, sex, race, education, BMI grade, smoking status, hypertension, diabetes, TG, TC, and HDL-C	8
Heredia et al. (2023) [41]	MAFLD, MASLD, moderate fibrosis, fibrosis	North America	USA	cross-sectional	187	age, sex, BMI, and kcal	7
Zhou et al. (2023) [42]	hepatocellular carcinoma, liver cancer	Asia	China	cohort	181,346	age, sex, race/ethnicity, education, diabetes, and hypertension	8
Xie et al. (2022) [43]	MAFLD, MASLD	Asia	China	cohort	66,526	age, sex, urbanicity, ethnicity, marital status, highest education attained, household income, profession, regular smoking, physical activity in metabolic equivalent tasks, total energy intake, regular intake of sweetened beverages, regular intake of dietary supplements, regular intake of spicy food, regular intake of pepper, insomnia symptoms, depressive symptoms, anxiety symptoms, menopause status for women, family history of cardiometabolic diseases, and BMI (models for DASH were additionally adjusted for alcohol intake)	7
Luu et al. (2021) [44]	hepatocellular carcinoma, liver cancer	Asia	China	cohort, case–control	91,603	age, sex, dialect, year of enrollment, education level, smoking status, coffee drinking status, alcohol drinking status, total energy intake, BMI, and diabetes status	8
Park et al. (2020) [45]	NAFLD, MASLD	North America	USA	cohort	5888	birth year, sex, race/ethnicity, length of Medicare enrollment, BMI, physical activity, total energy intake, and coffee consumption	9
Ma et al. (2019) [46]	hepatocellular carcinoma, liver cancer	North America	USA	cohort	173,229	age, race, cohort, physical activity level, body mass index, smoking, regular aspirin use, alcohol intake, total calorie intake, and type 2 diabetes	8
Bogumil et al. (2019) [47]	hepatocellular carcinoma, liver cancer	North America	USA	cohort	215,000	age at cohort entry, sex, race/ethnicity, BMI, history of diabetes, smoking status, and total energy	8
Maskarinec et al. (2017) [48]	NAFLD, MASLD	North America	USA	cohort	21,500	sex, age, ethnicity, total energy intake at cohort entry, and physical activity at cohort entry	8
Li et al. (2014) [49]	chronic liver disease	North America	USA	cohort	1053	age, sex, race, smoking, alcohol intake, education, BMI, diabetes, usual activity throughout the day, vigorous physical activity, and total energy intake	7

NOS: Newcastle–Ottawa Scale; NAFLD: nonalcoholic fatty liver disease; MAFLD: metabolic-associated fatty liver disease; MASLD: metabolic-associated steatohepatitis—liver disease; BMI: body mass index.

**Table 2:** Risk relationship of aMED for the prevention of chronic liver disease.

	No. of Studies	OR (95% CI)	*I* ^2^
Disease Type			
MASLD	6	0.86 (0.75, 0.99)	66.0%
liver cancer	7	0.69 (0.60, 0.79)	0.0%
chronic liver disease	6	0.52 (0.46, 0.58)	0.0%
fibrosis	1	0.48 (0.10, 2.29)	NA
Geography			
Asia	5	0.70 (0.53, 0.92)	80.6%
North America	10	0.67 (0.54, 0.83)	83.6%
Europe	5	0.54 (0.44, 0.66)	0.0%
Study Design			
cohort	15	0.66 (0.55, 0.78)	86.9%
case–control	2	0.47 (0.32, 0.69)	0.0%
cross-sectional	3	0.81 (0.68, 0.96)	0.0%
Sample Size			
≥1000	3	0.50 (0.33, 0.76)	0.0%
<1000	17	0.66 (0.57, 0.77)	85.4%

CI, confidence interval; OR, odds ratio; NA, not applicable.

## Data Availability

The original contributions of this study are available in the article and its Supplementary Material, which includes Table S1 (PRISMA 2020 Checklist). For further inquiries, please contact the corresponding author.

## Supplementary Materials

The following supporting information can be downloaded at https://www.mdpi.com/article/10.3390/nu17182914/s1: Table S1: PRISMA 2020 checklist.

## References

[1] Seto W.K., Mandell M.S. (2021). Chronic liver disease: Global perspectives and future challenges to delivering quality health care. PLoS ONE.

[2] Manikat R., Ahmed A., Kim D. (2024). Current epidemiology of chronic liver disease. Gastroenterol. Rep..

[3] Singal A.K. (2024). Alcohol-Associated Liver Disease: Treatment and Prevention. Clin. Liver Dis..

[4] Zeng X.F., Varady K.A., Wang X.D., Targher G., Byrne C.D., Tayyem R., Latella G., Bergheim I., Valenzuela R., George J. (2024). The role of dietary modification in the prevention and management of metabolic dysfunction-associated fatty liver disease: An international multidisciplinary expert consensus. Metab. Clin. Exp..

[5] Berumen J., Baglieri J., Kisseleva T., Mekeel K. (2021). Liver fibrosis: Pathophysiology and clinical implications. WIREs Mech. Dis..

[6] Aller de la Fuente R. (2022). Nutrition and Chronic Liver Disease. Clin. Drug Investig..

[7] Brunner K.T., Henneberg C.J., Wilechansky R.M., Long M.T. (2019). Nonalcoholic Fatty Liver Disease and Obesity Treatment. Curr. Obes. Rep..

[8] Takegoshi K., Honda M., Okada H., Takabatake R., Matsuzawa-Nagata N., Campbell J.S., Nishikawa M., Shimakami T., Shirasaki T., Sakai Y. (2017). Branched-chain amino acids prevent hepatic fibrosis and development of hepatocellular carcinoma in a non-alcoholic steatohepatitis mouse model. Oncotarget.

[9] Xu R., Zhang L., Pan H., Zhang Y. (2024). Retinoid X receptor heterodimers in hepatic function: Structural insights and therapeutic potential. Front. Pharmacol..

[10] Song J., Jiang Z.G. (2023). Low vitamin A levels are associated with liver-related mortality: A nationally representative cohort study. Hepatol. Commun..

[11] Gao Y., Zhang W., Zeng L.Q., Bai H., Li J., Zhou J., Zhou G.Y., Fang C.W., Wang F., Qin X.J. (2020). Exercise and dietary intervention ameliorate high-fat diet-induced NAFLD and liver aging by inducing lipophagy. Redox Biol..

[12] Ashrafizadeh M., Zarrabi A., Hushmandi K., Zarrin V., Moghadam E.R., Hashemi F., Makvandi P., Samarghandian S., Khan H., Hashemi F. (2020). Toward Regulatory Effects of Curcumin on Transforming Growth Factor-Beta Across Different Diseases: A Review. Front. Pharmacol..

[13] Li C., Zhang R., Zhan Y., Zheng J. (2021). Resveratrol Inhibits Hepatic Stellate Cell Activation via the Hippo Pathway. Mediat. Inflamm..

[14] Clugston R.D. (2020). Carotenoids and fatty liver disease: Current knowledge and research gaps. Biochim. Biophys. Acta Mol. Cell Biol. Lipids.

[15] Stice C.P., Xia H., Wang X.D. (2018). Tomato lycopene prevention of alcoholic fatty liver disease and hepatocellular carcinoma development. Chronic Dis. Transl. Med..

[16] Nishino A., Maoka T., Yasui H. (2021). Preventive Effects of β-Cryptoxanthin, a Potent Antioxidant and Provitamin A Carotenoid, on Lifestyle-Related Diseases-A Central Focus on Its Effects on Non-Alcoholic Fatty Liver Disease (NAFLD). Antioxidants.

[17] Kim M.N., Lo C.H., Corey K.E., Luo X., Long L., Zhang X., Chan A.T., Simon T.G. (2022). Red meat consumption, obesity, and the risk of nonalcoholic fatty liver disease among women: Evidence from mediation analysis. Clin. Nutr..

[18] Shan L., Wang F., Zhai D., Meng X., Liu J., Lv X. (2022). Caffeine in liver diseases: Pharmacology and toxicology. Front. Pharmacol..

[19] Liu L., Du J., Fan H., Yu Y., Luo Y., Gu F., Yu H., Liao X. (2024). Blueberry anthocyanins improve liver fibrosis by regulating NCOA4 ubiquitination through TRIM7 to affect ferroptosis of hepatic stellate cells. Am. J. Physiol. Gastrointest. Liver Physiol..

[20] Liu X., Baecker A., Wu M., Zhou J.Y., Yang J., Han R.Q., Wang P.H., Liu A.M., Gu X., Zhang X.F. (2019). Raw Garlic Consumption and Risk of Liver Cancer: A Population-Based Case-Control Study in Eastern China. Nutrients.

[21] Almatroodi S.A., Alsahli M.A., Almatroudi A., Rahmani A.H. (2019). Garlic and its Active Compounds: A Potential Candidate in The Prevention of Cancer by Modulating Various Cell Signalling Pathways. Anti-Cancer Agents Med. Chem..

[22] Wang W., Liu Y., Li Y., Luo B., Lin Z., Chen K., Liu Y. (2023). Dietary patterns and cardiometabolic health: Clinical evidence and mechanism. MedComm.

[23] Steck S.E., Murphy E.A. (2020). Dietary patterns and cancer risk. Nat. Rev. Cancer.

[24] Lu P.Y., Shu L., Shen S.S., Chen X.J., Zhang X.Y. (2017). Dietary Patterns and Pancreatic Cancer Risk: A Meta-Analysis. Nutrients.

[25] Zhao L., Kase B., Zheng J., Steck S.E. (2023). Dietary Patterns and Risk of Lung Cancer: A Systematic Review and Meta-Analyses of Observational Studies. Curr. Nutr. Rep..

[26] Feng Y.L., Shu L., Zheng P.F., Zhang X.Y., Si C.J., Yu X.L., Gao W., Zhang L. (2017). Dietary patterns and colorectal cancer risk: A meta-analysis. Eur. J. Cancer Prev. Off. J. Eur. Cancer Prev. Organ. (ECP).

[27] Chiavaroli L., Lee D., Ahmed A., Cheung A., Khan T.A., Blanco S., Mejia, Mirrahimi A., Jenkins D.J.A., Livesey G. (2021). Effect of low glycaemic index or load dietary patterns on glycaemic control and cardiometabolic risk factors in diabetes: Systematic review and meta-analysis of randomised controlled trials. BMJ.

[28] Toi P.L., Anothaisintawee T., Chaikledkaew U., Briones J.R., Reutrakul S., Thakkinstian A. (2020). Preventive Role of Diet Interventions and Dietary Factors in Type 2 Diabetes Mellitus: An Umbrella Review. Nutrients.

[29] Muñoz Fernández S.S., Lima Ribeiro S.M. (2018). Nutrition and Alzheimer Disease. Clin. Geriatr. Med..

[30] Ellouze I., Sheffler J., Nagpal R., Arjmandi B. (2023). Dietary Patterns and Alzheimer’s Disease: An Updated Review Linking Nutrition to Neuroscience. Nutrients.

[31] Śliwińska S., Jeziorek M. (2021). The role of nutrition in Alzheimer’s disease. Rocz. Panstw. Zakl. Hig..

[32] Venter C., Eyerich S., Sarin T., Klatt K.C. (2020). Nutrition and the Immune System: A Complicated Tango. Nutrients.

[33] Picone P., Girgenti A., Buttacavoli M., Nuzzo D. (2024). Enriching the Mediterranean diet could nourish the brain more effectively. Front. Nutr..

[34] Zeraattalab-Motlagh S., Jayedi A., Shab-Bidar S. (2022). Mediterranean dietary pattern and the risk of type 2 diabetes: A systematic review and dose-response meta-analysis of prospective cohort studies. Eur. J. Nutr..

[35] Agarwal S., Fulgoni V.L., Jacques P.F. (2022). Association of 100% Fruit Juice Consumption with Cognitive Measures, Anxiety, and Depression in US Adults. Nutrients.

[36] Mori K., Akiyama Y., Tanaka M., Sato T., Endo K., Hosaka I., Hanawa N., Sakamoto N., Furuhashi M. (2024). Deciphering metabolic dysfunction-associated steatotic liver disease: Insights from predictive modeling and clustering analysis. J. Gastroenterol. Hepatol..

[37] Page M.J., McKenzie J.E., Bossuyt P.M., Boutron I., Hoffmann T.C., Mulrow C.D., Shamseer L., Tetzlaff J.M., Akl E.A., Brennan S.E. (2021). The PRISMA 2020 statement: An updated guideline for reporting systematic reviews. BMJ.

[38] Huang X., Zhang X., Hao X., Wang T., Wu P., Shen L., Yang Y., Wan W., Zhang K. (2025). Association of dietary quality and mortality in the non-alcoholic fatty liver disease and advanced fibrosis populations: NHANES 2005–2018. Front. Nutr..

[39] Huang X., Gan D., Fan Y., Fu Q., He C., Liu W., Li F., Ma L., Wang M., Zhang W. (2024). The Associations between Healthy Eating Patterns and Risk of Metabolic Dysfunction-Associated Steatotic Liver Disease: A Case-Control Study. Nutrients.

[40] Li T., Zhao J., Cao H., Han X., Lu Y., Jiang F., Li X., Sun J., Zhou S., Sun Z. (2024). Dietary patterns in the progression of metabolic dysfunction-associated fatty liver disease to advanced liver disease: A prospective cohort study. Am. J. Clin. Nutr..

[41] Heredia N.I., Thrift A.P., Ramsey D.J., Loomba R., El-Serag H.B. (2023). Association of Diet Quality with Metabolic (Dysfunction) Associated Fatty Liver Disease in Veterans in Primary Care. Nutrients.

[42] Zhou K., Lim T., Dodge J.L., Terrault N.A., Wilkens L.R., Setiawan V.W. (2023). Population-attributable risk of modifiable lifestyle factors to hepatocellular carcinoma: The multi-ethnic cohort. Aliment. Pharmacol. Ther..

[43] Xie X., Guo B., Xiao X., Yin J., Wang Z., Jiang X., Li J., Long L., Zhou J., Zhang N. (2022). Healthy dietary patterns and metabolic dysfunction-associated fatty liver disease in less-developed ethnic minority regions: A large cross-sectional study. BMC Public Health.

[44] Luu H.N., Neelakantan N., Geng T.T., Wang R., Goh G.B., Clemente J.C., Jin A., van Dam R.M., Jia W., Behari J. (2021). Quality diet indexes and risk of hepatocellular carcinoma: Findings from the Singapore Chinese Health Study. Int. J. Cancer.

[45] Park S.Y., Noureddin M., Boushey C., Wilkens L.R., Setiawan V.W. (2020). Diet Quality Association with Nonalcoholic Fatty Liver Disease by Cirrhosis Status: The Multiethnic Cohort. Curr. Dev. Nutr..

[46] Ma Y., Yang W., Simon T.G., Smith-Warner S.A., Fung T.T., Sui J., Chong D., VoPham T., Meyerhardt J.A., Wen D. (2019). Dietary Patterns and Risk of Hepatocellular Carcinoma Among U.S. Men and Women. Hepatology.

[47] Bogumil D., Park S.Y., Le Marchand L., Haiman C.A., Wilkens L.R., Boushey C.J., Setiawan V.W. (2019). High-Quality Diets Are Associated With Reduced Risk of Hepatocellular Carcinoma and Chronic Liver Disease: The Multiethnic Cohort. Hepatol. Commun..

[48] Maskarinec G., Lim U., Jacobs S., Monroe K.R., Ernst T., Buchthal S.D., Shepherd J.A., Wilkens L.R., Le Marchand L., Boushey C.J. (2017). Diet Quality in Midadulthood Predicts Visceral Adiposity and Liver Fatness in Older Ages: The Multiethnic Cohort Study. Obesity.

[49] Li W.Q., Park Y., McGlynn K.A., Hollenbeck A.R., Taylor P.R., Goldstein A.M., Freedman N.D. (2014). Index-based dietary patterns and risk of incident hepatocellular carcinoma and mortality from chronic liver disease in a prospective study. Hepatology.

[50] Hutchins-Wiese H.L., Bales C.W., Porter Starr K.N. (2022). Mediterranean diet scoring systems: Understanding the evolution and applications for Mediterranean and non-Mediterranean countries. Br. J. Nutr..

[51] Fung T.T., McCullough M.L., Newby P.K., Manson J.E., Meigs J.B., Rifai N., Willett W.C., Hu F.B. (2005). Diet-quality scores and plasma concentrations of markers of inflammation and endothelial dysfunction. Am. J. Clin. Nutr..

[52] Yue Y., Yuan C., Wang D.D., Wang M., Song M., Shan Z., Hu F., Rosner B., Smith-Warner S.A., Willett W.C. (2022). Reproducibility and validity of diet quality scores derived from food-frequency questionnaires. Am. J. Clin. Nutr..

[53] He Y., Yang Y., Cheng P., Zhang W., Jia J., Ye D., Wang J. (2025). Association Between Dietary Inflammatory Index and NAFLD: A Cross-Sectional Study of the National Health and Nutrition Examination Survey. Mediat. Inflamm..

[54] Eslam M., Newsome P.N., Sarin S.K., Anstee Q.M., Targher G., Romero-Gomez M., Zelber-Sagi S., Wai-Sun Wong V., Dufour J.F., Schattenberg J.M. (2020). A new definition for metabolic dysfunction-associated fatty liver disease: An international expert consensus statement. J. Hepatol..

[55] European Association for the Study of the Liver, European Association for the Study of Diabetes (EASD) (2024). EASL-EASD-EASO Clinical Practice Guidelines on the management of metabolic dysfunction-associated steatotic liver disease (MASLD). J. Hepatol..

[56] Govaere O., Petersen S.K., Martinez-Lopez N., Wouters J., Van Haele M., Mancina R.M., Jamialahmadi O., Bilkei-Gorzo O., Lassen P.B., Darlay R. (2022). Macrophage scavenger receptor 1 mediates lipid-induced inflammation in non-alcoholic fatty liver disease. J. Hepatol..

[57] Guo Y., Zhu X., Zeng M., Qi L., Tang X., Wang D., Zhang M., Xie Y., Li H., Yang X. (2021). A diet high in sugar and fat influences neurotransmitter metabolism and then affects brain function by altering the gut microbiota. Transl. Psychiatry.

[58] Huang H.C., Lee P.N., Huang W.C., Yang H.Y. (2023). Partial Replacement of Diet with Dehulled Adlay Ameliorates Hepatic Steatosis, Inflammation, Oxidative Stress, and Gut Dysbiosis in Rats with Nonalcoholic Fatty Liver Disease. Nutrients.

[59] Romero-Gómez M., Zelber-Sagi S., Trenell M. (2017). Treatment of NAFLD with diet, physical activity and exercise. J. Hepatol..

[60] Willcox D.C., Willcox B.J., Todoriki H., Suzuki M. (2009). The Okinawan diet: Health implications of a low-calorie, nutrient-dense, antioxidant-rich dietary pattern low in glycemic load. J. Am. Coll. Nutr..

[61] Rodríguez-Negrete E.V., Morales-González Á., Madrigal-Santillán E.O., Sánchez-Reyes K., Álvarez-González I., Madrigal-Bujaidar E., Valadez-Vega C., Chamorro-Cevallos G., Garcia-Melo L.F., Morales-González J.A. (2024). Phytochemicals and Their Usefulness in the Maintenance of Health. Plants.

[62] Zhu Y., Ji X., Yuen M., Yuen T., Yuen H., Wang M., Smith D., Peng Q. (2021). Effects of Ball Milling Combined With Cellulase Treatment on Physicochemical Properties and in vitro Hypoglycemic Ability of Sea Buckthorn Seed Meal Insoluble Dietary Fiber. Front. Nutr..

[63] Zhang D.Y., Cheng D.C., Cao Y.N., Su Y., Chen L., Liu W.Y., Yu Y.X., Xu X.M. (2022). The effect of dietary fiber supplement on prevention of gestational diabetes mellitus in women with pre-pregnancy overweight/obesity: A randomized controlled trial. Front. Pharmacol..

[64] Margolis L.M., Rivas D.A., Ezzyat Y., Gaffney-Stomberg E., Young A.J., McClung J.P., Fielding R.A., Pasiakos S.M. (2016). Calorie Restricted High Protein Diets Downregulate Lipogenesis and Lower Intrahepatic Triglyceride Concentrations in Male Rats. Nutrients.

[65] Wang Y., Zhang Y., Wang Z., Yu L., Chen K., Xie Y., Liu Y., Liang W., Zheng Y., Zhan Y. (2022). The interplay of transcriptional coregulator NUPR1 with SREBP1 promotes hepatocellular carcinoma progression via upregulation of lipogenesis. Cell Death Discov..

[66] Adeola O.L., Agudosi G.M., Akueme N.T., Okobi O.E., Akinyemi F.B., Ononiwu U.O., Akunne H.S., Akinboro M.K., Ogbeifun O.E., Okeaya-Inneh M. (2023). The Effectiveness of Nutritional Strategies in the Treatment and Management of Obesity: A Systematic Review. Cureus.

[67] Barania Adabi S., Daneghian S., Khalkhali H., Nejadrahim R., Shivappa N. (2023). The association between inflammatory and immune system biomarkers and the dietary inflammatory index in patients with COVID-19. Front. Nutr..

[68] Lee S.W., Lee Y.J., Baek S.M., Kang K.K., Kim T.U., Yim J.H., Kim H.Y., Han S.H., Choi S.K., Park S.J. (2022). Mega-Dose Vitamin C Ameliorates Nonalcoholic Fatty Liver Disease in a Mouse Fast-Food Diet Model. Nutrients.

[69] Zhang W., Zhang Y., Wei M., Zhu K., Wang X., Zhao Y., Shi J., Liu Z. (2025). Global burden of TBL cancer in older adults: The role of dietary factors (1990–2021). BMC Public Health.

[70] Chen X., Sun L., Wang S., Wang Y., Zhou Y., Li Y., Cheng Z., Wang Y., Jiang Y., Zhao Z. (2023). Effects of Prunus Tomentosa Thumb Total Flavones on adjuvant arthritis in rats and regulation of autophagy. Technol. Health Care Off. J. Eur. Soc. Eng. Med..

[71] Sharma M., Kaur R., Kaushik K., Kaushal N. (2019). Redox modulatory protective effects of ω-3 fatty acids rich fish oil against experimental colitis. Toxicol. Mech. Methods.

[72] Zhuang C., Yuan J., Du Y., Zeng J., Sun Y., Wu Y., Gao X.H., Chen H.D. (2022). Effects of Oral Carotenoids on Oxidative Stress: A Systematic Review and Meta-Analysis of Studies in the Recent 20 Years. Front. Nutr..

[73] Zhu T., Chen X., Wang Q., Li F., Yang J., Zhu X., Wang J., Bo J. (2025). Causal Association Between 12 Micronutrients and Common Chronic Respiratory Diseases: A Bidirectional Two-Sample Mendelian Randomization Study. Genet. Res..

[74] Kyriakidou Y., Wood C., Ferrier C., Dolci A., Elliott B. (2021). The effect of Omega-3 polyunsaturated fatty acid supplementation on exercise-induced muscle damage. J. Int. Soc. Sports Nutr..

[75] Meital L.T., Windsor M.T., Perissiou M., Schulze K., Magee R., Kuballa A., Golledge J., Bailey T.G., Askew C.D., Russell F.D. (2019). Omega-3 fatty acids decrease oxidative stress and inflammation in macrophages from patients with small abdominal aortic aneurysm. Sci. Rep..

[76] Martins F.O., Conde S.V. (2022). Impact of Diet Composition on Insulin Resistance. Nutrients.

[77] Skeie G., Fadnes L.T. (2024). Cereals and cereal products—A scoping review for Nordic Nutrition Recommendations 2023. Food Nutr. Res..

[78] Liang J., Wen Y., Yin J., Zhu G., Wang T. (2024). Utilization of plant-based foods for effective prevention of chronic diseases: A longitudinal cohort study. npj Sci. Food.

[79] Vallazhath A., Thimmappa P.Y., Joshi H.B., Hebbar K.R., Nayak A., Umakanth S., Saoji A.A., Manjunath N.K., Hadapad B.S., Joshi M.B. (2025). A comprehensive review on the implications of Yogic/Sattvic diet in reducing inflammation in type 2 diabetes. Nutr. Diabetes.

[80] Smoak P., Burke S.J., Collier J.J. (2021). Botanical Interventions to Improve Glucose Control and Options for Diabetes Therapy. SN Compr. Clin. Med..

[81] Chen S., Li B., Chen L., Jiang H. (2023). Identification and validation of immune-related biomarkers and potential regulators and therapeutic targets for diabetic kidney disease. BMC Med. Genom..

[82] Lim G.H., Neelakantan N., Lee Y.Q., Park S.H., Kor Z.H., van Dam R.M., Chong M.F., Chia A. (2024). Dietary Patterns and Cardiovascular Diseases in Asia: A Systematic Review and Meta-Analysis. Adv. Nutr..

[83] Zhang Y., Yu W., Zhang L., Wang M., Chang W. (2022). The Interaction of Polyphenols and the Gut Microbiota in Neurodegenerative Diseases. Nutrients.

[84] Jahić Mujkić A., Tušek Žnidarič M., Berbić S., Žerovnik E. (2021). Synergy of the Inhibitory Action of Polyphenols Plus Vitamin C on Amyloid Fibril Formation: Case Study of Human Stefin B. Antioxidants.

[85] Guo X.F., Wang C., Yang T., Ma W.J., Zhai J., Zhao T., Xu T.C., Li J., Liu H., Sinclair A.J. (2022). The effects of fish oil plus vitamin D(3) intervention on non-alcoholic fatty liver disease: A randomized controlled trial. Eur. J. Nutr..

[86] Chen H.K., Lan Q.W., Li Y.J., Xin Q., Luo R.Q., Wang J.J. (2024). Association between Dietary Potassium Intake and Nonalcoholic Fatty Liver Disease and Advanced Hepatic Fibrosis in U.S. Adults. Int. J. Endocrinol..

